# Chicago Medical Society

**Published:** 1868-05-01

**Authors:** 


### *SOCIETY REPORTS.*

### CHICAGO MEDICAL SOCIETY.

At the regular annual meeting of the society, held in the Court House on the evening of the 3rd inst., the following officers were elected for the ensuing year:

*President*—Dr. C. Marguerat; *Vice-President*—Dr. R. G. Bogue; *Secretary and Treasurer*—Dr. P. S. Macdonald; *Board of Censors*—Drs. Wickersham, Reid, and Loverin; *Committee on Ethics*—Drs. Davis, Paoli, and Ross; *Sanitary Committee*—Drs. Hatch, Trimble, and Seely.

Delegates to the meeting of the American Medical Association, to be held at Washington on Tuesday, May 5th, 1868, were chosen as follows: Drs. R. C. Hamill, G. Reid, A. Fisher, G. C. Paoli, E. L. Holmes, and Rush.

The following were elected delegates to the meeting of the Illinois State Medical Society, to be held at Quincy on the third Tuesday in May, 1868: Drs. G. S. Hildreth, E. L. Holmes, Ira Hatch, T. D. Fitch, Thomas Bevan, R. G. Bogue, G. C. Paoli, N. T. Quales, John Guenin; G. M. Hutchinson, G. Reid, DeLaskie Miller, E. Marguerat, F. O. Earle.

It is said that a resolution was adopted instructing the delegates not to vote for any person as an officer, in either the State or American organization, who is not a member of a local society, if there is such an one in the county from which he hails.

